# COVID-19 and HIV-Associated Immune Reconstitution Inflammatory Syndrome: Emergence of Pathogen-Specific Immune Responses Adding Fuel to the Fire

**DOI:** 10.3389/fimmu.2021.649567

**Published:** 2021-03-24

**Authors:** Nabila Seddiki, Martyn French

**Affiliations:** ^1^ Inserm, U955, Equipe 16, Créteil, 94000, France, Université Paris Est, Faculté de Médecine, Créteil, France; ^2^ Vaccine Research Institute (VRI), Créteil, France; ^3^ School of Biomedical Sciences, University of Western Australia, Perth, WA, Australia; ^4^ Division of Immunology, PathWest Laboratory Medicine, Perth, WA, Australia

**Keywords:** COVID-19, immune reconstitution inflammatory syndrome, SARS-CoV-2, human immunodeficiency virus type 1, interleukin-18

## Abstract

Both coronavirus disease 2019 (COVID-19) and mycobacterial immune reconstitution inflammatory syndrome (IRIS) in patients with HIV-1 infection result from immunopathology that is characterized by increased production of multiple pro-inflammatory chemokines and cytokines associated with activation of myeloid cells (monocytes, macrophages and neutrophils). We propose that both conditions arise because innate immune responses generated in the absence of effective adaptive immune responses lead to monocyte/macrophage activation that is amplified by the emergence of a pathogen-specific adaptive immune response skewed towards monocyte/macrophage activating activity by the immunomodulatory effects of cytokines produced during the innate response, particularly interleukin-18. In mycobacterial IRIS, that disease-enhancing immune response is dominated by a Th1 CD4^+^ T cell response against mycobacterial antigens. By analogy, it is proposed that in severe COVID-19, amplification of monocyte/macrophage activation results from the effects of a SARS-CoV-2 spike protein antibody response with pro-inflammatory characteristics, including high proportions of IgG3 and IgA2 antibodies and afucosylation of IgG1 antibodies, that arises from B cell differentiation in an extra-follicular pathway promoted by activation of mucosa-associated invariant T cells. We suggest that therapy for the hyperinflammation underlying both COVID-19 and mycobacterial IRIS might be improved by targeting the immunomodulatory as well as the pro-inflammatory effects of the ‘cytokine storm’.

## Introduction

Infection with the novel coronavirus SARS-CoV-2 has very variable outcomes ranging from an asymptomatic infection through to coronavirus disease 2019 (COVID-19), which usually presents as respiratory tract disease ranging in severity from a flu-like illness to a severe viral pneumonia that may progress to acute respiratory distress syndrome (ARDS) and/or critical illness in about 20% of patients (1, 2). A coagulopathy is a prominent feature of critical illness in COVID-19 and contributes to morbidity and mortality (3). Deterioration of respiratory tract disease and progression to a critical state usually commences about 10 days after symptom onset (1). Risk factors for deterioration include older age, male sex and medical co-morbidities, such as obesity, diabetes mellitus and hypertension (1, 2). Children rarely develop respiratory tract disease caused by SARS-CoV-2 infection but may develop pediatric multisystem inflammatory syndrome (4).

It has become clear that most disease manifestations of SARS-CoV-2 infection are a consequence of hyperinflammation resulting from SARS-CoV-2-induced immunopathology, which is characterized by lymphopenia and neutrophilia (1, 2), increased production of multiple pro-inflammatory chemokines and cytokines detectable in plasma (5, 6) or broncho-alveolar lavage (BAL) fluid (7), and dysfunction and/or activation of myeloid cells (monocytes, macrophages and neutrophils), which has been demonstrated both in blood (8–10) and BAL fluid (11). Indeed, high plasma levels of D-dimers, ferritin, interleukin (IL)-6 and tumor necrosis factor-alpha (TNF-α) are strong predictors of mortality in COVID-19 (1, 6). Many of these abnormalities bear similarities to those observed in severely immunodeficient patients with human immunodeficiency virus type 1 (HIV-1) infection who develop an immune reconstitution inflammatory syndrome (IRIS) after commencing antiretroviral therapy (ART) consequent upon the restoration of an immune response against an opportunistic pathogen. Here, we compare the immunopathology underlying HIV-associated IRIS with that currently reported for COVID-19 and reason that new insights into the immunopathology underlying COVID-19 and treatment of it may arise from doing this.

## Mycobacterial IRIS Is a Manifestation of Myeloid Cell Activation Amplified by an Emergent Th1 CD4^+^ T Cell Response Against Mycobacterial Antigens

Approximately 20% of people with HIV-1 infection who commence ART with severe CD4^+^ T cell depletion (CD4^+^ T cell count <100/μL) experience an IRIS during the first 3 months of ART associated with a treated or unrecognized infection by various opportunistic pathogens (12). While clinical features of an IRIS may differ with different pathogens, an atypical and/or exaggerated inflammatory response is a characteristic finding. An IRIS may be associated with many types of mycobacteria, fungi, parasites and viruses that cause opportunistic infections but infections with mycobacteria are the most common cause, particularly *Mycobacterium tuberculosis* and *Mycobacterium avium* complex (MAC) (12). Research studies undertaken in patients with, or animal models of, mycobacterial IRIS have been most informative about the immunopathogenesis of IRIS and, therefore, mycobacterial IRIS will be focused on here. Notably, tuberculosis-associated IRIS (TB-IRIS) presents at a median time of 10-16 days after commencing ART (13–15).

Like COVID-19, increased production of multiple pro-inflammatory chemokines and cytokines is a prominent feature of mycobacterial IRIS (16–22), including increased production of TNF-α and IL-6. The latter has been shown to be a major mediator of immunopathology in animal models of MAC-IRIS (20). Furthermore, the IL-6-174*C variant of the IL-6 gene, as well as the TNFA-308*2 variant of the TNFA gene, were reported to be significantly less frequent in patients with mycobacterial IRIS compared with controls (23), suggesting a genetic susceptibility to the pro-inflammatory effects of IL-6 and TNF-α in mycobacterial IRIS. Corticosteroid therapy decreases the frequency and severity of TB-IRIS in patients at-risk of developing this condition (24) associated with suppressed production of pro-inflammatory cytokines, including IL-6 and TNF-α (18).

Patients with HIV infection who develop TB-IRIS also exhibit evidence of monocyte activation, not only after but also before commencing ART (19). In addition, higher blood neutrophil counts, increased neutrophil activation and elevation in plasma levels of neutrophil elastase and human neutrophil peptides have been observed in patients with TB-IRIS (25). Activation of circulating monocytes, and presumably tissue macrophages and neutrophils, is a likely explanation for increased plasma levels of pro-inflammatory cytokines and other biomarkers of inflammation being associated with an increased risk of developing TB-IRIS after ART is commenced. In a large prospective multinational study of patients with HIV-1 infection and CD4^+^ T cell counts <100/μL (12), Vinhaes et al. defined an inflammatory profile that predicted the development of mycobacterial IRIS, which included several biomarkers of monocyte/macrophage activation, including IL-6, TNF-α, IL-27, sCD14 and D-dimers (26). Monocyte activation in patients with TB-IRIS is also associated with NLRP3-inflammasome activation both before and after ART (27), which probably explains why high plasma IL-18 levels are also predictive of the development of TB-IRIS (16, 21). Furthermore, monocyte/macrophage activation is a likely explanation for observations that high plasma levels of sCD14, IL-6 and D-dimers are predictors of death after ART is commenced in people with HIV-1 infection (28, 29).

Current evidence suggests that following commencement of ART in patients with HIV-1 and mycobacterial infections, activation of monocytes, and presumably other myeloid cells, is amplified directly or indirectly by the restoration of CD4^+^ T cell responses against antigens of live or dead mycobacteria, resulting in an aberrant inflammatory response. Exaggerated and/or atypical inflammatory responses against mycobacteria, subsequently defined as mycobacterial IRIS, were first identified in patients with HIV-1 infection who were unable to generate T cell responses against mycobacterial antigens, assessed by measuring tuberculin skin test (TST) responses, until shortly after the commencement of ART (30, 31). Analyses of T cell responses using various laboratory methods in patients who developed mycobacterial IRIS after commencing combination ART have demonstrated an association with expansion of mycobacterial-specific T cells (13, 22, 32–35) with skewing of CD4^+^ T cells towards a Th1 phenotype during immune reconstitution (33–36), commensurate with the original observations of an association between mycobacterial IRIS and development of TST responses (30, 31). As IL-18 strongly promotes Th1 T cell responses (37), increased IL-18 production resulting from monocyte/macrophage activation (16, 18, 21, 27) may contribute to Th1 skewing of CD4^+^ T cells during immune reconstitution in patients with TB-IRIS. Furthermore, proliferation of weakly suppressive regulatory CD4^+^ T cells (38) may lead to inadequate regulation of T cell responses against mycobacteria in MAC-IRIS, though a relationship with decreased regulatory T cell numbers has not clearly been shown in TB-IRIS (33).

## Hyperinflammation in COVID-19 May be a Manifestation of Myeloid Cell Activation Amplified by a Pro-inflammatory Antibody Response Against SARS-CoV-2

While there is robust evidence that the hyperinflammation complicating SARS-CoV-2 infection is a consequence of myeloid cell dysfunction and/or activation (8–11) resulting in the increased production of multiple pro-inflammatory cytokines and chemokines (5, 6), sometimes referred to as a cytokine storm (39), mechanisms remain unclear. A pro-inflammatory innate immune response associated with ineffective type I interferon anti-viral activity (40), which in some patients may be caused by neutralizing autoantibodies to type I interferons (41), may be a contributing factor. However, uncertainty remains as to why the onset of hyperinflammation is about 10 days after the onset of SARS-CoV-2 infection symptoms, and why older age, male sex and medical co-morbidities such as diabetes mellitus increase the risk of developing severe COVID-19.

Reports that critical illness in COVID-19 is associated with higher serum levels of IgG and IgA antibodies to SARS-CoV-2 spike protein (SP) (42–45), have raised the possibility that antibody responses against SARS-CoV-2 might be a determinant of the immunopathology in patients with COVID-19. Notably, patients with severe COVID-19 usually deteriorate about 10 days after symptom onset (1) just after IgG antibodies to SARS-CoV-2 SP are detectable (43). Furthermore, Cervia et al. reported that very high serum levels of IgA antibodies to SARS-CoV-2 SP are highly predictive of ARDS (44). Higher serum levels of SARS-CoV-2 SP antibodies in patients with severe COVID-19 might reflect higher SARS-CoV-2 viral loads (46) and/or an uncoordinated adaptive immune response that includes impaired T cell responses against SARS-CoV-2. Thus, several studies have demonstrated that patients with severe COVID-19, when compared to patients with mild COVID-19, exhibit less robust CD4^+^ and CD8^+^ T cell responses against SARS-CoV-2 membrane, nucleocapsid, and spike proteins (47–49). However, the findings of studies undertaken in non-human primates to elucidate the immunopathology of SARS-CoV-1 infection have provided evidence that binding of antibody-coated viruses to activatory Fc gamma receptors (FcγRs) on macrophages might have a disease-enhancing effect. Thus, induction of antibodies to SARS-CoV-1 SP by vaccination, or passive immunization with SARS-CoV-1 SP IgG antibodies, was associated with pulmonary inflammation after animals were infected by SARS-CoV-1 (50). This inflammation was characterized by “skewing’’ of pulmonary macrophages towards a pro-inflammatory M1 phenotype. Furthermore, incubation of M2 macrophages from healthy humans with plasma from SARS patients and SARS-CoV-1 pseudoviruses induced production of IL-8, CCL2 and IL-6 (50).

Evidence that IgG antibodies to the SP of SARS-CoV-2 might also exert a pro-inflammatory effect on monocytes/macrophages in patients with severe COVID-19 has been provided from several sources. In a very comprehensive analysis of antibody responses against SARS-CoV-2 undertaken by Zohar et al. (51), it was observed that patients with severe COVID-19 (requiring admission to an intensive care unit), when compared to patients with moderate COVID-19, possessed IgG antibodies at 2 weeks after presentation that exhibited functional characteristics likely to induce monocyte/macrophage activation. Those characteristics included higher serum levels of antibodies to the SARS-CoV-2 SP, including the receptor binding domain (RBD), belonging to the IgG3 subclass, the most pro-inflammatory IgG subclass (52), greater antibody binding to FcγRIIa, FcγRIIb, FcγRIIIa and FcγRIIIb, and greater antibody-dependent phagocytosis and NK cell activating activity. However, most of these differences were not observed in patients with severe COVID-19 who subsequently died suggesting that additional factors are determinants of mortality. Chakraborty et al. (53), also demonstrated that functional characteristics of IgG antibodies to the RBD domain of SARS-CoV-2 SP may be more important than serum antibody levels. Specifically, severe COVID-19 was associated with a SARS-CoV-2 RBD IgG antibody response that exhibited a higher proportion of IgG3 antibodies and decreased fucosylation of the Fc region glycans of IgG1 antibodies, both of which are antibody characteristics associated with increased binding of immune complexes to activatory FcγRs. These findings were confirmed in an independent study that demonstrated decreased fucosylation of SARS-CoV-2 SP IgG antibodies in COVID-19 patients with ARDS when compared to patients without ARDS (54). Chakraborty et al. (53) also demonstrated that decreased antibody fucosylation was associated with higher binding of immune complexes to FcγRIIIa and increased production of the pro-inflammatory cytokines IL-6, TNF-α and IL-1β from monocytes incubated with immune complexes of antibodies and SARS-CoV-2 pseudoviruses. Notably, decreased antibody fucosylation did not appear to be directly related to the severity of SARS-CoV-2 infection but was more common in males with severe COVID-19. However, factors other than sex probably contributed to production of afucosylated antibodies because the association with male sex was not observed in patients with mild COVID-19.

Aberrant glycosylation of IgG1 Fc glycans is a consequence of B cell activation and/or metabolic activity driven by various extracellular stimuli, which include pro-inflammatory cytokines (55, 56). Such pro-inflammatory cytokines produced during an innate immune response against SARS-CoV-2 might include IL-6 because it promotes B cell differentiation (57). However, other cytokines are likely to contribute, including IL-18 because it plays a critical role in IgG antibody responses produced from B cells co-stimulated by subcapsular medullary macrophages in mice (58) and human B cells constitutively express IL-18R (59) but not IL-6R (60). Emerging evidence suggests that a pro-inflammatory antibody response in patients with severe COVID-19 may be derived from B cells that have differentiated into antibody secreting cells through an extrafollicular pathway (extrafollicular B cells). These cells are more abundant than normal in the blood of patients with severe COVID-19 (61, 62), as well as in lymphoid tissue, where they are increased in the context of marked germinal center depletion and impaired differentiation of germinal center T follicular helper cells (62). B cells that have differentiated through this pathway form a subpopulation of ‘double negative’ (DN; IgD^-^ CD27^-^) B cells, which are characterized by the immunophenotype CD11c^+^, CXCR5^-^, CXCR3^+^, T-bet^hi^ (type 2 DN [DN2] B cells) and programmed to differentiate through this pathway by IFN-γ in a TLR7-dependent manner (63). Immunoglobulin isotype switching of DN2 B cells in other infectious diseases is skewed towards pro-inflammatory isotypes of IgG, particularly IgG3 (64, 65). In the context of an early immune response against SARS-CoV-2, a major source of IFN-γ is likely to be mucosa-associated invariant T (MAIT) cells as these cells are activated and substantially depleted from blood in patients with severe COVID-19 (66, 67) and, furthermore, a fatal outcome of COVID-19 was associated with higher production of IFN-γ, compared with other cytokines, by MAIT cells (67). MAIT cells contribute to immune responses against viruses in an IL-18 dependent manner (68) and, importantly, MAIT cell activation was particularly associated with high plasma IL-18 levels in patients with severe COVID-19 (67). Th1 CD4^+^ T cells, which are increased in frequency in lymphoid tissue of patients with severe COVID-19 (62), might also be a source of IFN-γ.

A notable finding from a study of sex differences in immune responses in patients with COVID-19 was that while all patients exhibited increased production of multiple pro-inflammatory cytokines, male patients exhibited higher production of IL-18, as well as IL-8, associated with greater activation of non-classical monocytes, when compared with female COVID-19 patients (69). Furthermore, in a study of a small number of children with pediatric multisystem inflammatory syndrome or COVID-19, plasma IL-18 levels were increased in addition to IL-6 levels (70). Moreover, Rodrigues et al. demonstrated that severe COVID-19 was associated with NLRP3-inflammasome activation in blood mononuclear cells and monocytes from post-mortem tissues, and that serum IL-18 levels correlated with disease severity (71). Increased IL-18 production and MAIT cell activation during an innate immune response against SARS-CoV-2 may therefore contribute to B cell activation through an extra-follicular pathway and production of a SARS-CoV-2 antibody response with pro-inflammatory characteristics, which include skewing towards IgG3 antibodies and decreased fucosylation of IgG1 antibodies to SARS-CoV-2 SP.

Higher serum levels of SARS-CoV-2 IgA antibodies, particularly those of the pro-inflammatory IgA2 subclass (72), and the greater binding of IgA antibodies to FcαR, reported in severe COVID-19 patients compared with moderate COVID-19 patients at 2 weeks after presentation (51), might also contribute to activation of alveolar macrophages *via* Fc alpha receptors (73). Finally, skewing of SARS-CoV-2 SP IgG antibody responses towards IgG3 antibody production in patients with severe COVID-19 (51, 53) might also adversely affect immune responses against SARS-CoV-2 by mechanisms other than myeloid cell activation. Combes et al. demonstrated that patients with severe COVID-19 produced SARS-CoV-2 antibodies that block the production of interferon-stimulated genes in several cell-types by activating conserved signaling circuits that dampen cellular responses to interferons (74). In other situations, this effect is associated with an anti-viral IgG antibody response that consists of IgG3 as well as IgG1 antibodies (75).

## Implications for Therapy of COVID-19

Suppression of inflammatory responses by the use of corticosteroid therapy is at least partially effective in the prevention and treatment of TB-IRIS (18, 24) and treatment of severe COVID-19 (76). However, corticosteroid therapy may be complicated by opportunistic infections, such as Kaposi’s sarcoma, in TB-IRIS (77) and potentially may further impair T cell responses against SARS-CoV-2. Anti-inflammatory therapies that target particular pro-inflammatory cytokines, such as TNF-α and IL-6, have been shown to be effective treatment for mycobacterial IRIS in studies of small numbers of patients (78) but observational studies and randomized controlled trials have not provided clear evidence of a benefit of IL-6 inhibitors, such as the IL-6R blocker tocilizumab, in the treatment of severe COVID-19 (79–81). An alternative or additional approach to controlling inflammation in severe COVID-19, as well as mycobacterial IRIS, is to modulate the effects of the cytokine milieu arising from the initial innate immune response that, as suggested here, might be a determinant of the functional characteristics of emerging adaptive immune responses. For example, investigating the effects of inhibiting IL-18 activity with humanized monoclonal antibodies to IL-18 (82), or suppression of NLRP3-inflammasome activation, is supported by preliminary data from uncontrolled, but more than one, clinical studies in patients with COVID-19 reporting a beneficial effect of inhibiting NLRP3-inflammasome activity with colchicine (83, 84).

## Summary and Conclusions

**Figure 1:**
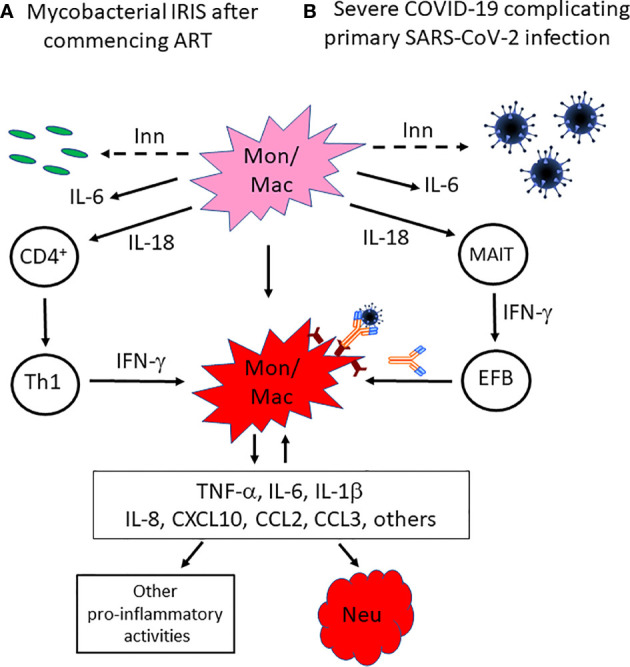
Proposed mechanisms by which innate immune responses not only induce inflammation but also skew emerging adaptive immune responses towards a response that amplifies myeloid cell activation in mycobacterial IRIS or COVID-19. In the absence of effective adaptive immune responses against **(A)** mycobacteria in HIV patients with severe CD4^+^ T cell deficiency and a mycobacterial co-infection, or **(B)** acute infection with the novel pathogen SARS-CoV-2, innate immune responses are generated that result in activation of monocytes/macrophages, including NLRP3-inflammasome activation. While this does not control the mycobacterial or SARS-CoV-2 infection, it generates a pro-inflammatory cytokine environment that includes IL-18, which skews CD4^+^ T cell recovery towards a Th1 response after ART is commenced in HIV patients, or induces MAIT cell activation that promotes B cell differentiation through an extra-follicular pathway and the production of a SARS-CoV-2 SP antibody response with pro-inflammatory characteristics, including decreased fucosylation of IgG1 Fc glycans and higher proportions of IgG3 and IgA2 antibodies, which enhances binding of SARS-CoV-2/antibody complexes to activatory FcγRs on macrophages, in COVID-19 patients. In either situation, dysregulated production and activity of multiple pro-inflammatory cytokines and chemokines occurs and has multiple effects, including further recruitment and activation of neutrophils and monocytes, as well as macrophage activation in a positive feedback loop. Inn, innate immune response; Mon/mac, monocytes/macrophages; Neu, neutrophils; MAIT, mucosa-associated invariant T cells; EFB, extra-follicular B cells.

While COVID-19 is a complication of acute SARS-CoV-2 infection and HIV-associated IRIS occurs in the context of chronic HIV-1 infection, similarities between the two conditions are apparent (**Table 1**) and have been considered here in order to enlighten the immunopathology of severe COVID-19. Approximately 20% of HIV patients with severe CD4^+^ T cell deficiency develop an IRIS after commencing ART, which presents at a median time of 10-16 days for TB-IRIS, while approximately 20% of people with symptomatic SARS-CoV-2 infection develop severe COVID-19 at about 10 days after symptom onset. We suggest that, in the absence of adaptive immune responses resulting from severe CD4^+^ T cell depletion caused by HIV-1 infection, or infection with a novel pathogen in people with SARS-CoV-2 infection, innate immune responses against mycobacteria or SARS-CoV-2, respectively, are induced leading to activation of monocytes/macrophages, including NLRP3-inflammasome activation and IL-18 production. When adaptive immune responses emerge, through ART-induced immune reconstitution in people with HIV-1 infection or production of a primary antibody response in patients with SARS-CoV-2 infection, they directly or indirectly amplify the activation of monocytes/macrophages and neutrophils resulting in an exaggerated inflammatory response and immunopathology in infected tissues (**Figure 1**). In addition, we suggest that the initial innate immune response not only primes the immune system for hyperinflammation induced by emergent adaptive immune responses but also exerts an immunomodulatory effect on those adaptive immune responses resulting in a Th1-skewed CD4^+^ T cell response against mycobacteria in mycobacterial IRIS and a SARS-CoV-2 IgG antibody response with pro-inflammatory characteristics, arising from extra-follicular B cell differentiation promoted by MAIT cell activation, in COVID-19. Based on our comparison of the immunopathology underlying mycobacterial IRIS and COVID-19, we suggest that IL-18 may play a central role in this process and should be investigated further as a possible therapeutic target.

**Table 1:** Similarities between HIV-associated mycobacterial IRIS and severe COVID-19.

	HIV-associated mycobacterial IRIS	Severe COVID-19
Time of disease onset	Median time of 10-16 days after commencing ART (TB-IRIS)	Approximately 10 days after symptom onset
Markers of monocyte/macrophage activation associated with an increased risk of disease and death	sCD14, D-dimers	sCD14, D-dimers, ferritin
Increased plasma levels of pro-inflammatory cytokines and chemokines associated with disease	IL-6, IL-8, IL-12, IL-18, TNF-α, IFN-γ, CXCL10	IL-1β, IL-6, IL-8, IL-12, IL-17, IL-18, IFN-γ, TNF-α, CCL2, CCL3, CXCL10, GM-CSF
Emergent adaptive immune response that may amplify monocyte/macrophage activation associated with disease onset	Mycobacteria-specific Th1 CD4^+^ T cell response	SARS-CoV-2 SP antibody response with pro-inflammatory characteristics

## Data Availability Statement

The original contributions presented in the study are included in the article/supplementary material. Further inquiries can be directed to the corresponding author.

## Author Contributions

MF conceived the hypothesis outlined in this paper, wrote the manuscript, and produced the figure. NS wrote the manuscript, produced the figure, and obtained funding for publication. All authors contributed to the article and approved the submitted version.

## Funding

Publication of this work was supported by the Investissement d’Avenir program managed by the ANR under reference ANR-10-LABX-77, the Agence Nationale pour la Recherche sur le SIDA et les hépatites virales (ANRS), and the Vaccine Research Institute (VRI).

## Conflict of Interest

The authors declare that the research was conducted in the absence of any commercial or financial relationships that could be construed as a potential conflict of interest.
